# Multi-center evaluation of the Alinity m HR HPV assay with liquid-based cytology cervical specimens in the United States

**DOI:** 10.1128/spectrum.01918-24

**Published:** 2025-01-27

**Authors:** D. Yitzchak Goldstein, Tong Yang, Danijela Lucic, Yan Zhang, Richard Cullum, Joshua Kostera, Anami Patel

**Affiliations:** 1 Montefiore Medical Center, Bronx, New York, USA; 2 Ochsner Health, New Orleans, Louisiana, USA; 3 Molecular Diagnostics of Abbott, Des Plaines, Illinois, USA; 4 PathAI Diagnostics, Memphis, Tennessee, USA; Emory University School of Medicine, Atlanta, Georgia, USA

**Keywords:** human papillomavirus, cervical cancer, liquid-based cytology, risk stratification

## Abstract

Incorporating molecular testing for human papillomavirus (HPV) into the screening of cervical specimens can improve risk stratification and, in turn, patient management. Infection with a high-risk (HR) HPV genotype is associated with greater risk for persistent infection, viral integration, and progression of cervical neoplasia. Current guidelines consider HPV 16 or HPV 18 clinically actionable with referral to colposcopy; however, 12 Other HR HPV genotypes have been associated with cervical cancer risk, suggesting a benefit of extended genotyping. In this multi-center study, we evaluated the performance of the Alinity m HR HPV assay, which reports HPV 16, 18, and 45 individually and aggregates of HPV 31/33/52/58 and HPV 35/39/51/56/59/66, compared with cobas HPV and Aptima HPV assays, across a variety of cytology result categories. A total of 746 de-identified residual cervical specimens, collected as part of routine cervical cancer screening programs, were tested using Alinity m HR HPV and at least one comparator assay. The overall percent agreement was ≥90.7% for results from the Alinity m HR HPV assay and cobas HPV assays and 90.5% for results from the Alinity m HR HPV and Aptima HPV assay. In patients with any abnormal cytology result, Alinity m identified 78 specimens with non-HPV 16/18 results, underscoring the benefit of detecting additional HR HPV genotypes to guide patient management more accurately. Among specimens with normal cytology, Alinity m detected 14 additional specimens with non-HPV 16/18 genotypes. Extended HR HPV testing can provide additional information to triage patients for appropriate testing and follow-up.

**IMPORTANCE:**

Extended genotyping for high-risk human papillomavirus (HPV) types enhances diagnostic precision by identifying additional oncogenic HPV types beyond 16 and 18 therefore offering a more nuanced risk profile. This more comprehensive detection may aid in identifying persistent infections that are more likely to progress, thereby supporting future risk-based patient management strategies.

## INTRODUCTION

Nearly all cervical cancers can be attributed to persistent infection with human papillomavirus (HPV). HPV infection has an estimated prevalence of 40%, and nearly 24 million new infections were diagnosed in persons 15–59 years of age in 2018 (1). HPV is a diverse and ubiquitous group of DNA viruses that belong to the family *Papillomaviridae*; a subset of 14 high-risk (HR) HPV types (16, 18, 31, 33, 35, 39, 45, 51, 52, 56, 58, 59, 66, and 68) within the *Alphapapillomavirus* genus infect mostly mucosal and genital regions and are associated with cancer (2). Most HPV infections are innocuous with a majority being immunologically cleared within 12 months. Persistent infection with HR HPV types, however, is recognized as a significant risk factor for cervical pathogenesis leading to transformation of the cervical epithelium, resulting in cervical neoplasia (3, 4). Infection with one or more of the HR HPV genotypes is associated with invasive cervical cancer or precancers. Three HR HPV genotypes, 16, 18, and 45, have been reported to account for 75% of squamous cell carcinoma and 94% of adenocarcinoma cases in younger women (5). While detection of either HPV 16 or 18 in cervical specimens is considered clinically actionable, studies have shown a close relationship between infection with HPV 31, 33, 52, or 58 and a significant risk of high-grade cervical carcinogenesis (6–8). The Alinity m HR HPV assay (Abbott Molecular, Des Plaines, IL) was designed to stratify HPV 16, 18, and 45 individually and aggregates reporting of HPV 31, 33, 52, and 58 into Other HR HPV A and HPV 35, 39, 51, 56, 59, 66, and 68 into Other HR HPV B groups.

The Alinity m HR HPV assay received CE mark in 2019 and has been used in several clinical studies outside of the United States (US) to evaluate the performance of the Alinity assay compared to other CE-marked HPV assays (9–11). The Alinity m HR HPV assay received FDA approval in October 2023. Here, we describe the first real-world clinical evaluations of the Alinity m HR HPV assay in the US. We examined the clinical performance of the Alinity m HR HPV assay to detect HR HPV in routine liquid-based cytology (LBC) cervical specimens at three geographically diverse sites in the US. The sites included one commercial reference laboratory (PathAI Diagnostics [PAD], Memphis, TN) and two large academic medical centers (Montefiore Medical Center [MMC], Bronx, NY and Ochsner Medical Center [OMC], New Orleans, LA). PAD compared the performance of the Alinity m HR HPV assay to the Hologic Aptima HPV assay (Marlborough, MA) and Roche cobas 6800 HPV assay (Basel, Switzerland). MMC and OMC compared the performance of the Alinity m HR HPV assay to the cobas 4800 HPV assay (Roche). An extended analysis included overall percent agreement (OPA) between the comparator assays for the three study sites and a composite comparator analysis at PAD.

## MATERIALS AND METHODS

### Specimens and setting

This multi-center study included a total of 746 de-identified residual cervical clinical specimens collected by clinicians as part of routine cervical cancer screening. A total of 274 specimens were included from PAD, 323 specimens from MMC, and 149 specimens from OMC. In order to ensure HPV genotype representation across the study sites, specimen inclusion criteria varied at each study site and depended on institutional policy and/or preference. PAD preselected positive and negative specimens based on cytology and HPV genotype. OMC preselected positive and negative specimens based on cytology and HPV positivity. Initially, MMC tested all specimens, then enriched the study population for specimens positive for HPV16 and HPV18 genotypes.

Specimens were collected in LBC media (ThinPrep PreservCyt Solution [Hologic] or SurePath Solution [BD, Franklin Lakes, NJ]) and processed at each site. PAD specimens were processed for cytology using the ThinPrep 2000 (TP 2000) system, MMC specimens were processed for cytology using the BD Totalys SlidePrep, and OMC specimens were processed using the TP 2000 system. Each site aliquoted 1 mL of specimen for HPV testing. At PAD and MMC, the study was considered exempt from institutional review board (IRB) approval, as all identifying information was removed from the specimens prior to testing for this study. At OMC, all patient identifiers were removed from the specimens, and the study was conducted in accordance with an approved Ochsner Health Institutional Review Board protocol.

### Cytology

Cytology results were interpreted using the 2014 Bethesda System (12). Specimens were classified based on cytology as negative for intraepithelial lesion (NILM) or any atypical cytology (≥ASC-US), which included atypical squamous cells of undetermined significance (ASC-US), atypical glandular cells (AGC), atypical squamous cells cannot rule out high-grade lesion (ASC-H), low-grade squamous intraepithelial lesion (LSIL), high-grade intraepithelial lesion (HSIL), and adenocarcinoma.

### Assays

The cobas HPV test for use on the cobas 4800 system (cobas 4800) and the cobas HPV test for use on the cobas 6800 system (cobas 6800) are automated qualitative PCR-based tests that detect the 14 HR HPV genotypes (differentiating between HPV 16, HPV 18, and Other HR HPV [31/33/35/39/45/51/52/56/58/59/66/68]) using primers and probes targeting the L1 region of the HPV genome (13, 14). Both cobas tests include beta-globin as a cellular control for each specimen and utilize external positive and negative assay controls. The cobas 4800 test is FDA-approved for use with clinician-collected cervical specimens placed in ThinPrep PreservCyt Solution or SurePath Preservative Fluid. Specimens collected in SurePath fluid must be pretreated prior to processing on the cobas 4800. Briefly, 0.5 mL of a SurePath sample is diluted in 0.5 mL of cobas Sample Prep Buffer and heated for 20 minutes at 120°C. After heating, the diluted specimens are cooled at ambient temperature for 10 minutes before being loaded onto the cobas 4800 for processing. The cobas 6800 test is FDA approved for use with clinician-collected cervical specimens placed in ThinPrep only. Specimens are prepared for amplification and detection using automated nucleic acid extraction and purification performed by the cobas 4800 and 6800 systems. In this study, specimens were run on the cobas 6800 platform at PAD and on the cobas 4800 platform at MMC and OMC.

The Aptima HPV assay (Aptima) is a qualitative transcription-mediated amplification (TMA) test that detects E6/E7 mRNA for the 14 HR HPV genotypes in cervical specimens (15). It does not distinguish between the various HR HPV genotypes. The Aptima assay is FDA approved for cervical specimens collected in ThinPrep PreservCyt Solution. Prior to processing specimens using the Aptima assay, 1.0 mL of collected specimens must be transferred to a tube containing specimen transport media for cell lysis and mRNA stabilization. Once loaded on the Hologic Panther System, prepared specimens undergo automated target capture, target amplification by TMA, and detection of amplicon by a hybridization protection assay. The Aptima assay utilizes an exogenous internal control as a process control and external positive and negative assay calibrators. The Aptima assay was run on the Panther system at PAD.

The Alinity m HR HPV assay (Alinity m) for use on the Alinity m System is a qualitative real-time PCR-based assay that simultaneously amplifies and detects genotypes HPV 16, HPV 18, and HPV 45 while reporting the 11 other HR HPV genotypes in two aggregates: HR HPV Other A (31/33/52/58) and HR HPV Other B (35/39/51/56/59/66/68) (16). The assay uses primer and probes targeting the L1 region of the HPV genome for detection and differentiation of HPV genotypes. The assay also includes beta-globin as a cellular control to ensure specimen adequacy and sample extraction and amplification efficiency. In addition to the cellular control, the assay uses external positive and negative controls ran every 24 hours. The Alinity m assay is FDA approved for cervical specimens collected in ThinPrep PreservCyt Solution or SurePath Preservative Fluid. An aliquot (0.5–2.0 mL) of specimens collected in ThinPrep or SurePath must be transferred to a transport tube before processing on the Alinity m System. Specimens loaded on the Alinity m System, undergo automated sample preparation, nucleic acid extraction, PCR assembly, amplification/detection, and result calculation and reporting. In this study, ThinPrep specimens were processed on the Alinity m at PAD and OMC, and SurePath specimens were processed on the Alinity m at MMC.

### Precision and reproducibility

Precision and reproducibility were evaluated at PAD (ThinPrep) and MMC (SurePath) using commercially available panels from Microbix (Mississauga, Ontario, Canada) that included HPV panels VP-62-M1 (16/18/45), VP-62-M2 (31/33/66), and VP-62-M3 (39/51/52). Panels were handled and stored according to the package insert and thawed at room temperature prior to immediate use. Panels VP-62-M1 and VP-62-M2 were each diluted 1:1 by aliquoting 1 mL of material from the panel vial to 1 mL of either ThinPrep or SurePath in an Alinity transport tube and mixed, and 0.60 mL were distributed to each of three Alinity transport tubes then placed on the Alinity m system and run with the Alinity m HR HPV assay. Panel VP-62-M3 was not diluted; panel vial contents were mixed and aliquoted to an Alinity transport tube then placed on the Alinity m system and run with the Alinity m HR HPV assay. These panels were repeated in the same manner over 3 consecutive days.

### Statistical analysis

Percent agreement between results from the Alinity m HR HPV assay and each comparator assay was determined for all specimens. OPA was calculated between the assays at all three study sites. A composite comparator analysis was performed using the data from PAD since each specimen had a result from the cobas 6800, Aptima, and Alinity m assay. For the composite comparator analysis, a specimen was considered a “true” positive if at least two of the assays reported a positive result for the specimen. Likewise, a specimen was considered a “true” negative if at least two of the assays reported a negative result for the specimen. Once “true” positive and negative results were assigned to each specimen from PAD, percent agreement values were calculated for each assay compared to the “true” results. All data analyses were performed using SAS software version 9.3 or higher (SAS, Cary, NC).

## RESULTS

**Fig 1:**
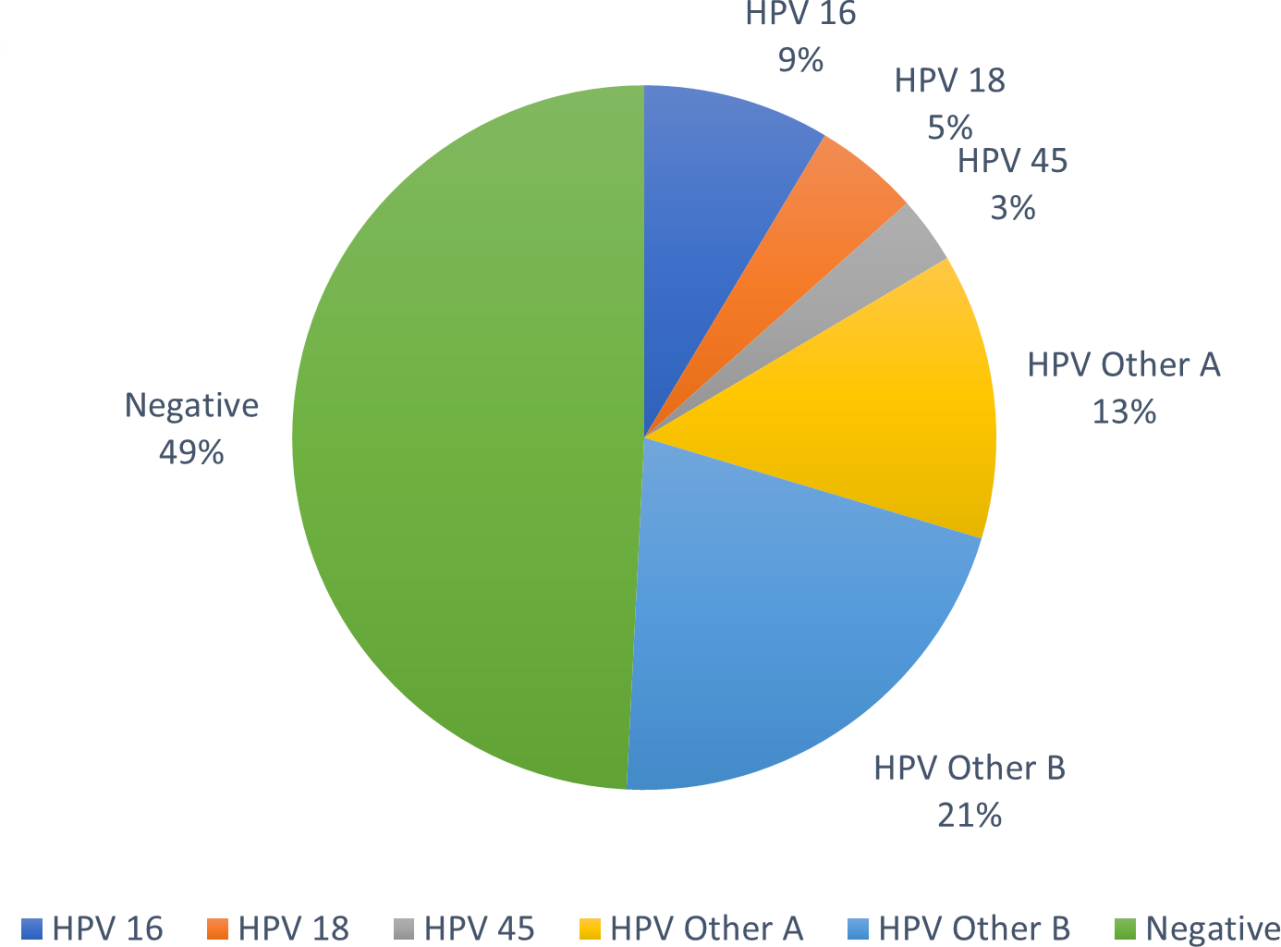
Alinity m HR HPV result distribution for all specimens by percentage across all three sites.

At PAD, of the 274 specimens tested, 37.2% (102/274) were NILM by cytology, and 62.8% (172/274) were ≥ASC-US. At MMC, 80.2% (259/323) were NILM by cytology, and 19.8% (64/323) were ≥ASC-US. At OMC, 37.6% (56/149) were NILM by cytology, and 62.4% (93/149) were ≥ASC-US. The distribution of cytology results and Alinity m HR HPV assay results at each study site is shown in Table 1 (PAD), Table 2 (MMC), and Table 3 (OMC), and Alinity m HR HPV result distribution across all sites regardless of cytology is shown graphically in Fig. 1.

**TABLE 1:** PAD cytology result distribution by hierarchical HPV genotype reported by the Alinity m HR HPV assay^*a*^

Cytology result	HPV16	HPV18	HPV45	Other HR HPV A	Other HR HPV B	HR HPV detected	Not detected	Total
ASC-US	7	5	4	18	35	69	51	120
ASC-H	0	0	1	0	0	1	1	2
AGC	0	0	0	0	0	0	1	1
LSIL	3	2	1	7	18	31	12	43
HSIL	3	0	0	2	1	6	0	6
≥ASC-US	13	7	6	27	54	107	65	172
NILM	0	0	0	1	6	7	95	102
Total	13	7	6	28	60	114	160	274

^*a*^ AGC, atypical glandular cells; ASC-H, atypical squamous cells cannot rule out high-grade lesion; ASC-US, atypical squamous cells of undetermined significance; HSIL, high-grade intraepithelial lesion; HPV, human papillomavirus; LSIL, low-grade squamous intraepithelial lesion; NILM, negative for intraepithelial lesion.

**TABLE 2:** MMC cytology result distribution by HPV genotype reported by the Alinity m HR HPV assay^*a*^

Cytology result	HPV16	HPV18	HPV45	Other HR HPV A	Other HR HPV B	HR HPV detected	Not detected	Total
ASC-US	4	3	2	12	6	27	7	34
ASC-H	1	0	0	0	1	2	0	2
AGC	0	0	0	1	0	1	0	1
LSIL	4	4	2	1	3	14	5	19
LSIL-H	0	0	2	0	1	3	0	3
HSIL	3	0	0	0	0	3	0	3
SIL-H	2	0	0	0	0	2	0	2
≥ASC-US	14	7	6	14	11	52	12	64
NILM	31	18	3	29	39	120	139	259
Total	45	25	9	43	50	172	151	323

^*a*^ AGC, atypical glandular cells; ASC-H, atypical squamous cells cannot rule out high-grade lesion; ASC-US, atypical squamous cells of undetermined significance; HSIL, high-grade intraepithelial lesion; HPV, human papillomavirus; LSIL, low-grade squamous intraepithelial lesion; NILM, negative for intraepithelial lesion.

**TABLE 3:** OMC cytology result distribution by hierarchical HPV genotype reported by the Alinity m HR HPV assay^*a*^

Cytology result	HPV16	HPV18	HPV45	Other HR HPV A	Other HR HPV B	HR HPV detected	Not detected	Total
Adenocarcinoma	0	0	0	0	0	0	1	1
AGC	0	0	0	0	2	2	2	4
ASC-US	2	1	0	11	23	37	19	56
HSIL	1	0	3	0	0	4	0	4
LSIL	2	2	3	8	10	25	3	28
≥ASC-US	5	3	6	19	35	68	25	93
NILM	1	1	2	8	13	25	31	56
Total	6	4	8	27	48	93	56	149

^*a*^ AGC, atypical glandular cells; ASC-US, atypical squamous cells of undetermined significance; HSIL, high-grade intraepithelial lesion; HPV, human papillomavirus; LSIL, low-grade squamous intraepithelial lesion; NILM, negative for intraepithelial lesion.

### Overall agreement between the Alinity m HR HPV, cobas HPV, and Aptima HPV assays

For PAD, OPA across all cytological categories was calculated for HR HPV Detected by the Alinity m assay vs cobas 6800 and Aptima assays, as well as cobas 6800 vs Aptima. The OPA was 93.8% (257/274) for Alinity m vs cobas 6800 and 90.5% (248/274) for Alinity m vs Aptima. OPA for cobas 6800 vs Aptima was 90.9% (249/274; Table 4). For MMC, the OPA across all cytological categories for HR HPV Detected by the Alinity m assay vs cobas 4800 assay was 90.7% (293/323; Table 5). For OMC, the OPA across all cytological categories for HR HPV Detected between the Alinity m assay vs cobas 4800 assay was 96.0% (143/149; Table 6).

**TABLE 4:** PAD OPA of Alinity m, cobas 6800, and Aptima results (ThinPrep)^*a*^

OPA	Alinity Result	Cobas 6800 result		
		Positive	Negative	Total
93.8 (257/274)	HR HPV Detected	108	6	114
	Not Detected	11	149	160
	Total	119	155	274
		Aptima Result		
OPA	Alinity Result	Positive	Negative	Total
90.5 (248/274)	HR HPV Detected	97	17	114
	Not Detected	9	151	160
	Total	106	168	274
		Aptima result		
OPA	cobas 6800 Result	Positive	Negative	Total
90.9 (249/274)	Positive	100	19	119
	Negative	6	149	155
	Total	106	168	274

^*a*^ HR HPV, high-risk human papillomavirus; OPA, overall percent agreement; PAD, PathAI Diagnostics.

**TABLE 5:** MMC OPA of Alinity m and cobas 4800 results (SurePath)^*a*^

OPA	Alinity Result	Cobas 4800 result		
		Positive	Negative	Total
90.7 (293/323)	HR HPV detected	155	17	172
	Not detected	13	138	151
	Total	168	155	323

^*a*^ HR HPV, high-risk human papillomavirus; MMC, Montefiore Medical Center; OPA, overall percent agreement.

**TABLE 6:** OMC OPA of Alinity m and cobas 4800 results (ThinPrep)^*a*^

OPA	Alinity Result	Cobas 4800 result		
		Positive	Negative	Total
96.0 (143/149)	HR HPV detected	91	2	93
	Not detected	4	52	56
	Total	95	54	149

^*a*^ HR HPV, high-risk human papillomavirus; OMC, Ochsner Medical Center; OPA, overall percent agreement.

### Precision and reproducibility

Precision and reproducibility were evaluated at PAD (ThinPrep) and MMC (SurePath) using commercially available panels from Microbix that included panels VP-62-M1 (16/18/45), VP-62-M2 (31/33/66), and VP-62-M3 (39/51/52). At each site, the panels were tested in triplicate over 3 days with three operators using one lot of Alinity m sample extraction and amplification reagents. Intra-Run CV% and Inter-Run CV% are presented in Table S1 (MMC) and Table S2 (PAD). Across all panels and both sites, we observed Intra-Run CV% ≤ 3.88% and Inter-Run CV% ≤ 3.40%

### Composite comparator agreement between the Alinity m HR HPV, cobas 6800 HPV, and Aptima HPV assays

Specimen testing at PAD included two HPV comparator assays for which a composite comparator analysis could be completed. For all specimens in the PAD cohort, Alinity m and the composite comparator results demonstrated a PPA of 95.7% (110/115). This finding was not statistically different from PPA for the cobas 6800 HPV assay vs composite comparator (98.3% [113/115]; *P*-value for the two-sided Fisher’s Exact test >0.05) but was notably different than the calculated PPA for the Aptima HPV assay vs composite comparator (88.7% [102/115]; *P*-value for the two-sided Fisher’s Exact test <0.05). Alinity m and cobas PPA values were higher than that of Aptima. The NPA calculated for Alinity m vs the composite comparator was the same as Aptima at 97.5% (155/159); this value was not statistically significantly different than the cobas 6800 HPV NPA (96.2% [153/159]; Table 7; *P*-value for the two-sided Fisher’s Exact test >0.05). In Table 7, PPA and NPA along with the two-sided 95% CI using Wilson score method were calculated.

**TABLE 7:** Composite comparator (CC) results for Alinity m, cobas 6800, and Aptima^*a*^

Category	*N*	PPA (%)		NPA (%)	
		Estimate	*n*/*N*	Estimate	*n*/*N*
		(95% CI)		(95% CI)	
Alinity vs CC	274	95.7 (90.2, 98.1)	110/115	97.5 (93.7, 99.0)	155/159
Cobas vs CC	274	98.3 (93.9, 99.5)	113/115	96.2 (92.0, 98.3)	153/159
Aptima vs CC	274	88.7 (81.6, 93.3)	102/115	97.5 (93.7, 99.0)	155/159

^*a*^ NPA, negative percent agreement; PPA, positive percent agreement.

## DISCUSSION

In this multi-center study, we found OPA ≥90.5% for results from the Alinity m HR HPV assay and cobas HPV assay run on either the cobas 4800 or 6800 platform and an OPA of 90.5% for results from the Alinity m HR HPV assay vs Aptima HPV assay. Higher overall agreement was observed between Alinity m HR HPV assay and cobas 6800 (93.8%) than between Alinity m HR HPV assay and Aptima HPV assay (90.5%; Table 4). Lower overall agreement was also observed between cobas 6800 and Aptima HPV assay (90.9%; Table 4). For the cobas 4800, we did observe a 5.3% difference in OPA between OMC (96.0% OPA) and MMC (90.7% OPA). This difference is largely due to the specimen inclusion criteria at the respective study sites. As noted previously, OMC preselected known HPV-positive and -negative samples for correlation. MMC, on the other hand, included all collected specimens for a period before enriching for HPV-positive specimens. The specimen collection method at MMC resulted in many near- and sub-limit of detection HPV-positive specimens being included in the correlation study which led to an increase in discordance compared to OMC. The findings, here, are consistent with a study performed in Slovenia by Oštrbenk et al. that demonstrated that the Alinity m HR HPV assay had comparable clinical performance to the cobas 4800 HPV assay (17). In the VALGENT-3 framework study, the Alinity m HR HPV assay was found to have non-inferior clinical accuracy compared to three other clinically validated HPV assays (9), and it fulfills international consensus guideline criteria for clinical use (11). The results of the composite comparator analysis from PAD were consistent with data from the CLEAR trial which demonstrated that the Aptima assay has a slightly lower sensitivity compared to an FDA-approved HPV DNA test (15). Overall, the concordance observed between the HPV testing platforms in this study was similar to previous US clinical trials comparing HPV tests (13–16).

A total of 329 specimens had cytology results that were ≥ASC-US, representing approximately 44.1% (329/746) of the study cohort. An ASC-US cytological result is the most common abnormal cervical cytology classification with variable clinical significance; it is suggestive of a squamous extraepithelial lesion but lacks criteria for conclusive interpretation (18). In patients with ≥ASC-US cytological results, Alinity m identified a total of 18 specimens with HPV 45 results and 62 specimens with Other HR HPV A (HPV 31, 33, 52, and 58) results across the three study sites with ≥ASC-US cytology. These specimens would be classified as “Other HPV” by the cobas HPV assay and “Detected” by the Aptima assay. Current management guidelines recommend referral to colposcopy with HPV16 or HPV18 positive results. HPV 18 and HPV 45 together are significantly more common in cases of adenocarcinoma (12%–32%) than squamous cell carcinoma (5%–8%) (5). Recent reports have categorically identified the need to stratify high-risk genotypes to further triage patients beyond the pooled detection of the 14 high-risk HPV genotypes for primary screening or co-testing with cytology to guide patient management (19). Alinity m HR HPV Other HR A genotypes (31/33/52/58) are responsible for 15% of cervical cancers and 11% of all HPV-associated cancers (20). HPV types 16, 18, 45, 33, 58, 52, and 31 contribute to 90% of all cervical cancers, and Alinity m HR HPV Other HR B genotypes (51/35/39/56/59/66/68) contribute to less than 9% of all cervical cancers (5).

Co-infections with multiple HR HPV types are a common finding of many molecular epidemiological and diagnostic investigations, with some studies suggesting a possible role in the progression of cervical neoplasia (3). In this study, we observed increased rates of HPV co-infection for specimens with ≥ASC-US cytology compared to NILM cytology. In the PAD study cohort, the Alinity m HR HPV assay co-infection rate was approximately 4.2% (5/120) and 14% (6/43) of samples with ASC-US and LSIL cytology results, respectively, and none for the other cytology categories. At MMC, the co-infection rate was 3.5% (9/259) in NILM, 5.9% (2/34) in ASC-US, and 23.3% (7/30) in >ASC-US samples. OMC had co-infection rates of 1.79% (1/56) in NILM, 1.79% (1/56) in ASC-US, 7.12% (2/28) in LSIL, and 25.0% (1/4) in HSIL samples, consistent with data reported elsewhere (3, 21). Notably, co-infections have been observed more frequently among younger women and those with abnormal cytology results where age-adjusted odds ratios showed a significant positive association between multiple infections and the presence of LSIL and HSIL compared to NILM (22). However, the cumulative effects of infection with multiple HR HPV genotypes on cervical carcinogenesis risk have not been clearly delineated.

Patients with a positive HR HPV test result with normal or NILM cytology results make up the majority of cases in the cervical screening population (15, 16). Those with HPV results are typically triaged based on HPV 16 or HPV 18 positivity in accordance with current guidance (5).

This study included 102 specimens at PAD with NILM cytology results; Alinity m detected six additional specimens with HPV compared to Aptima (one Other HR HPV A and five Other HR HPV B) and six additional specimens with HPV compared to cobas 6800 (one Other HR HPV A and five Other HR HPV B). At MMC, Alinity m detected nine additional specimens with HPV with NILM cytology compared to cobas 4800 (six HPV 16 and three HPV 18). Alinity m detected two additional specimens with HPV (Other HR HPV B) compared to cobas 4800 with NILM cytology results at OCM. It is worth noting that patients with NILM cytology results but positive for non-16/18 HPV are still at risk of developing precancerous lesions and cancer beyond 3 or 5 years. Furthermore, women who have persistent HPV infections with positive HPV test results and NILM cytology tend to develop cervical lesions of concern within a shorter interval than the recommended follow-up testing in current guidance (23).

Limitations of the study include the lack of biopsy/histology results for determining patient infection status and comparing assay accuracies and predictive values (10). Although histologic analysis was not available for the subjects in this cohort, previous studies evaluated the use of similar genotyping in women with ASC-US and found that referring only those with HPV 16, 18, and Alinity Other HR HPV A genotypes (31/33/52/58) to colposcopy reduced colposcopy referrals by 37% at the expense of delaying detection of 8% of ≥CIN2 lesions (24). A separate analysis from a different data set stratified by two additional age groups, ≥25 years (HPV primary screening population) and ≥30 years (co-testing population), showed similar results (25).

In this study, we found that the Alinity m HR HPV assay demonstrated higher clinical sensitivity than the Aptima HPV assay across all cytological categories, reflected in the PPA vs the composite comparator analysis, and had similar clinical sensitivity when compared with the cobas 6800 HPV assay for detecting HR HPV (Table 7). The Alinity m HR HPV assay also demonstrated good concordance across all cytological categories for detecting HR HPV compared with the cobas 4800 and 6800 assays (≥90.5%). Cervical cancer screening with HR HPV testing alone or with cytology is reported to be more sensitive than cytology alone in detecting squamous intraepithelial lesions of concern (8, 14). Extended HR HPV testing can provide additional information to triage patients for appropriate testing and follow-up.

## SUPPLEMENTAL MATERIAL

Supplemental tables spectrum.01918-24-s0001.pdf Tables S1 and S2.

ASM does not own the copyrights to Supplemental Material that may be linked to, or accessed through, an article. The authors have granted ASM a non-exclusive, world-wide license to publish the Supplemental Material files. Please contact the corresponding author directly for reuse.

## References

[1] Lewis RM, Laprise J-F, Gargano JW, Unger ER, Querec TD, Chesson HW, Brisson M, Markowitz LE. 2021. Estimated prevalence and incidence of disease-associated human papillomavirus types among 15- to 59-year-olds in the United States. Sex Transm Dis 48:273–277. doi:10.1097/OLQ.0000000000001356 33492097 PMC10037549

[2] Bamford DH, Zuckerman M. 2021. Encyclopedia of virology. 4th ed, p 493–501. Academic Press.

[3] Trottier H, Mahmud S, Costa MC, Sobrinho JP, Duarte-Franco E, Rohan TE, Ferenczy A, Villa LL, Franco EL. 2006. Human papillomavirus infections with multiple types and risk of cervical neoplasia. Cancer Epidemiol Biomarkers Prev 15:1274–1280. doi:10.1158/1055-9965.EPI-06-0129 16835323

[4] de Villiers E-M, Fauquet C, Broker TR, Bernard H-U, zur Hausen H. 2004. Classification of papillomaviruses. Virology (Auckl) 324:17–27. doi:10.1016/j.virol.2004.03.033 15183049

[5] de Sanjose S, Quint WG, Alemany L, Geraets DT, Klaustermeier JE, Lloveras B, Tous S, Felix A, Bravo LE, Shin H-R, et al.. 2010. Human papillomavirus genotype attribution in invasive cervical cancer: a retrospective cross-sectional worldwide study. Lancet Oncol 11:1048–1056. doi:10.1016/S1470-2045(10)70230-8 20952254

[6] Curry SJ, Krist AH, Owens DK, Barry MJ, Caughey AB, Davidson KW, Doubeni CA, Epling JW Jr, Kemper AR, Kubik M, Landefeld CS, Mangione CM, Phipps MG, Silverstein M, Simon MA, Tseng C-W, Wong JB, US Preventive Services Task Force. 2018. Screening for cervical cancer: US preventive services task force recommendation statement. JAMA 320:674–686. doi:10.1001/jama.2018.10897 30140884

[7] So KA, Lee IH, Lee KH, Hong SR, Kim YJ, Seo HH, Kim TJ. 2019. Human papillomavirus genotype-specific risk in cervical carcinogenesis. J Gynecol Oncol 30:e52. doi:10.3802/jgo.2019.30.e52 31074234 PMC6543103

[8] Hansen BT, Campbell S, Nygård M. 2018. Long-term incidence trends of HPV-related cancers, and cases preventable by HPV vaccination: a registry-based study in Norway. BMJ Open 8:e019005. doi:10.1136/bmjopen-2017-019005 PMC5855252 29476028

[9] Dhillon SK, Oštrbenk Valenčak A, Xu L, Poljak M, Arbyn M. 2021. Clinical and analytical evaluation of the Alinity m HR HPV assay within the VALGENT-3 Framework. J Clin Microbiol 59:e00286-21. doi:10.1128/JCM.00286-21 33731413 PMC8316144

[10] Jang D, Ratnam S, Smieja M, Speicher DJ, Arias M, Clavio A, Costescu D, Elit L, Huang S, Herrero-Garcia E, Joseph AM, Jiang H, Needle R, Chernesky M. 2021. Comparison of Alinity m HPV and cobas HPV assays on cervical specimens in diverse storage media. Tumour Virus Res 12:200224. doi:10.1016/j.tvr.2021.200224 34242835 PMC8319351

[11] Oštrbenk Valenčak A, Šterbenc A, Seme K, Poljak M. 2019. Alinity m HR HPV assay fulfills criteria for human papillomavirus test requirements in cervical cancer screening settings. J Clin Microbiol 58:e01120-19. doi:10.1128/JCM.01120-19 31666369 PMC6935899

[12] Nayar R, Wilbur DC. 2015. The Bethesda system for reporting cervical cytology. 3rd ed. Springer International. 10.1159/000477556 28693017

[13] cobas HPV test [package insert]. 2020. Branchburg, NJ Roche Molecular Systems, Inc

[14] cobas 6800 [system specifications]. 2015. Pleasanton, CA Roche Molecular Systems, Inc

[15] Aptima HPV assay [package insert]. 2011. Hologic, Inc San Diego, CA

[16] Alinity m HR HPV Assay [package insert]. 2022. Des Plaines, IL Abbott Molecular, Inc

[17] Oštrbenk Valenčak A, Bertram A, Gröning A, Poljak M. 2021. Comparison of the clinical and analytical performance of Alinity m HR HPV and cobas 4800 HPV assays in a population-based screening setting. J Clin Virol 140:104851. doi:10.1016/j.jcv.2021.104851 34020361

[18] Tewari R, Chaudhary A. 2010. Atypical squamous cells of undetermined significance: a follow up study. Med J Armed Forces India 66:225–227. doi:10.1016/S0377-1237(10)80042-5 27408306 PMC4921249

[19] Stoler MH, Baker E, Boyle S, Aslam S, Ridder R, Huh WK, Wright TC Jr. 2020. Approaches to triage optimization in HPV primary screening: extended genotyping and p16/Ki-67 dual-stained cytology-retrospective insights from ATHENA. Int J Cancer 146:2599–2607. doi:10.1002/ijc.32669 31490545 PMC7078939

[20] Meites E, Gee J, Unger E, Markowitz L. 2021. Human papillomavirus

[21] Monsonego J, Cox JT, Behrens C, Sandri M, Franco EL, Yap P-S, Huh W. 2015. Prevalence of high-risk human papilloma virus genotypes and associated risk of cervical precancerous lesions in a large U.S. screening population: data from the ATHENA trial. Gynecol Oncol 137:47–54. doi:10.1016/j.ygyno.2015.01.551 25667973

[22] Schmitt M, Depuydt C, Benoy I, Bogers J, Antoine J, Arbyn M, Pawlita M, VALGENT Study Group. 2013. Multiple human papillomavirus infections with high viral loads are associated with cervical lesions but do not differentiate grades of cervical abnormalities. J Clin Microbiol 51:1458–1464. doi:10.1128/JCM.00087-13 23447632 PMC3647930

[23] Lazare C, Xiao S, Meng Y, Wang C, Li W, Wang Y, Chen G, Wei J, Hu J, Xue M, Wu P. 2019. Evaluation of cervical intraepithelial neoplasia occurrence following the recorded onset of persistent high-risk human papillomavirus infection: a retrospective study on infection duration. Front Oncol 9:976. doi:10.3389/fonc.2019.00976 31632909 PMC6779720

[24] Lee B, Suh DH, Kim K, No JH, Kim YB. 2015. Utility of human papillomavirus genotyping for triage of patients with atypical squamous cells of undetermined significance by cervical cytology. Anticancer Res 35:4197–4202. 26124378

[25] Wright TC, Stoler MH, Parvu V, Yanson K, Cooper C, Andrews J. 2019. Risk detection for high-grade cervical disease using Onclarity HPV extended genotyping in women, ≥21 years of age, with ASC-US or LSIL cytology. Gynecol Oncol 154:360–367. doi:10.1016/j.ygyno.2019.05.012 31160073

