# Supplementary material for: Multi-center evaluation of the Alinity m HR HPV assay with liquid-based cytology cervical specimens in the United States
Source: Microbiol Spectr. 2025 Jan 27;13(3):e01918-24. doi: 10.1128/spectrum.01918-24 (PMC11878003; doi:10.1128/spectrum.01918-24)
Supplement: Supplemental tables — Tables S1 and S2. [file spectrum.01918-24-s0001.pdf]

Supplemental Materials

Supplemental Table 1. Alinity m HR HPV Precision / Reproducibility – MMC (SurePath)*

Sample ID	Run1/Day 1				Run 2/Day 2				Run 3/Day 3				Inter-Run CV%		
	Operator	1			Operator	2			Operator	3					
	Final Result	Target 1 CN[a]	Target 2 CN[a]	Target 3 CN[a]	Final Result	Target 1 CN[a]	Target 2 CN[a]	Target 3 CN[a]	Final Result	Target 1 CN[a]	Target 2 CN[a]	Target 3 CN[a]	Target 1	Target 2	Target 3
VP-62-M1 (HPV 16/18/45)	HPV16; HPV18; HPV45	25.08	22.18	25.34	HPV16; HPV18; HPV45	25.43	22.66	25.95	HPV16; HPV18; HPV45	26.37	22.87	26.35	1.41%	1.52%	1.30%
	HPV16; HPV18; HPV45	25.55	22.55	25.98	HPV16; HPV18; HPV45	25.71	22.77	25.84	HPV16; HPV18; HPV45	25.82	22.94	26.23			
	HPV16; HPV18; HPV45	25.86	22.29	26.47	HPV16; HPV18; HPV45	25.50	21.92	25.77	HPV16; HPV18; HPV45	25.44	22.35	26.00			
**Intra-Run CV%**		**1.54%**	**0.85%**	**2.19%**		**0.57%**	**2.06%**	**0.35%**		**1.81%**	**1.42%**	**0.68%**			
VP-62-M2 (HPV 31/33/66)	Other HR HPV A; Other HR HPV B	24.08	22.58	n/a	Other HR HPV A; Other HR HPV B	23.71	21.42	n/a	Other HR HPV A; Other HR HPV B	24.52	22.29	n/a	1.57%	2.17%	n/a
	Other HR HPV A; Other HR HPV B	24.07	22.03	n/a	Other HR HPV A; Other HR HPV B	24.02	21.51	n/a	Other HR HPV A; Other HR HPV B	24.33	21.91	n/a			
	Other HR HPV A; Other HR HPV B	24.49	22.26	n/a	Other HR HPV A; Other HR HPV B	24.98	22.94	n/a	Other HR HPV A; Other HR HPV B	23.96	22.18	n/a			
**Intra-Run CV%**		**0.99%**	**1.24%**	**n/a**		**2.73%**	**3.88%**	**n/a**		**1.17%**	**0.88%**	**n/a**			
VP-62-M3 (HPV 39/51/52)	Other HR HPV A; Other HR HPV B	23.25	21.71	n/a	Other HR HPV A; Other HR HPV B	23.29	21.49	n/a	Other HR HPV A; Other HR HPV B	23.06	21.48	n/a	1.61%	1.32%	n/a
	Other HR HPV A; Other HR HPV B	23.04	21.48	n/a	Other HR HPV A; Other HR HPV B	23.56	21.55	n/a	Other HR HPV A; Other HR HPV B	23.03	21.33	n/a			
	Other HR HPV A; Other HR HPV B	23.16	21.63	n/a	Other HR HPV A; Other HR HPV B	23.82	21.99	n/a	Other HR HPV A; Other HR HPV B	22.48	20.94	n/a			
**Intra-Run CV%**		**0.46%**	**0.54%**	**n/a**		**1.13%**	**1.26%**	**n/a**		**1.43%**	**1.31%**	**n/a**			
Intra-Run %	100%				100%				100%				100% Precision		

*Microbix panels VP-62-M1 and VP-62-M2 were diluted 1:1 in SurePath prior to running on the Alinity m System. VP-62-M3 was not diluted prior to running on Alinity m.

[a]CN = cycle number

Supplemental Table 2. Alinity m HR HPV Precision / Reproducibility – PAD (ThinPrep)*

Sample ID	Run1/Day 1				Run 2/Day 2				Run 3/Day 3				Inter-Run CV%		
	Operator	1			Operator	2			Operator	3					
	Final Result	Target 1 CN[a]	Target 2 CN[a]	Target 3 CN[a]	Final Result	Target 1 CN[a]	Target 2 CN[a]	Target 3 CN[a]	Final Result	Target 1 CN[a]	Target 2 CN[a]	Target 3 CN[a]	Target 1	Target 2	Target 3
VP-62-M1 (HPV 16/18/45)	HPV16; HPV18; HPV45	24.88	21.98	26.86	HPV16; HPV18; HPV45	24.29	21.37	26.26	HPV16; HPV18; HPV45	24.33	20.93	26.89	1.56%	2.34%	1.43%
	HPV16; HPV18; HPV45	25.13	22.28	26.98	HPV16; HPV18; HPV45	24.28	21.67	26.08	HPV16; HPV18; HPV45	24.13	20.82	26.66			
	HPV16; HPV18; HPV45	24.31	21.00	26.28	HPV16; HPV18; HPV45	23.96	21.16	25.93	HPV16; HPV18; HPV45	24.09	21.19	26.45			
**Intra-Run CV%**		**1.70%**	**3.08%**	**1.40%**		**0.78%**	**1.20%**	**%**		**0.53%**	**0.91%**	**0.83%**			
VP-62-M2 (HPV 31/33/66)	Other HR HPV A; Other HR HPV B	24.38	23.90	n/a	Other HR HPV A; Other HR HPV B	23.88	23.39	n/a	Other HR HPV A; Other HR HPV B	23.43	22.83	n/a	1.57%	1.56%	n/a
	Other HR HPV A; Other HR HPV B	24.17	23.86	n/a	Other HR HPV A; Other HR HPV B	23.96	23.22	n/a	Other HR HPV A; Other HR HPV B	23.99	23.73	n/a			
	Other HR HPV A; Other HR HPV B	23.31	23.15	n/a	Other HR HPV A; Other HR HPV B	24.32	23.76	n/a	Other HR HPV A; Other HR HPV B	23.64	23.38	n/a			
**Intra-Run CV%**		**2.37%**	**1.79%**	**n/a**		**0.97%**	**1.18%**	**n/a**		**1.19%**	**1.95%**	**n/a**			
VP-62-M3 (HPV 39/51/52)	Other HR HPV A; Other HR HPV B	24.53	23.92	n/a	Other HR HPV A; Other HR HPV B	24.40	23.63	n/a	Other HR HPV A; Other HR HPV B	24.07	23.10	n/a	2.02%	2.30%	n/a
	Other HR HPV A; Other HR HPV B	25.30	24.49	n/a	Other HR HPV A; Other HR HPV B	24.04	23.18	n/a	Other HR HPV A; Other HR HPV B	24.22	23.18	n/a			
	Other HR HPV A; Other HR HPV B	25.39	24.55	n/a	Other HR HPV A; Other HR HPV B	24.85	23.88	n/a	Other HR HPV A; Other HR HPV B	24.73	24.02	n/a			
**Intra-Run CV%**		**1.89%**	**1.43%**	**n/a**		**1.66%**	**1.51%**	**n/a**		**1.42%**	**2.17%**	**n/a**			
**Intra-Run %**	**100%**				**100%**				**100%**				**100% Precision**		

*Microbix panels VP-62-M1 and VP-62-M2 were diluted 1:1 in ThinPrep prior to running on the Alinity m System. VP-62-M3 was not diluted prior to running on Alinity m.

[a]CN = cycle number
